# El Consultor Homœopatico

**Published:** 1890-03

**Authors:** 


### BOOK NOTICES.

### El Consultor Homœopatico.

With the month of December, 1889, comes the last issue of *El Consultor Homœopatico*, of Barcelona. We mourn its decease, but as of old, at king's funerals. The Consultor is dead. Viva! *La Revista Homœopatica*, the journal wherein it will live anew!

